# The progressive elevation of alpha fetoprotein for the diagnosis of hepatocellular carcinoma in patients with liver cirrhosis

**DOI:** 10.1186/1471-2407-7-28

**Published:** 2007-02-08

**Authors:** Oscar Arrieta, Bernardo Cacho, Daniela Morales-Espinosa, Ana Ruelas-Villavicencio, Diana Flores-Estrada, Norma Hernández-Pedro

**Affiliations:** 1 Department of Medical Oncology, Instituto Nacional de Cancerología (INCan), Mexico City, Mexico; 2 Department of Internal Medicine, Instituto Nacional de Ciencias Médicas y Nutrición "Salvador Zubirán" (INCMNSZ), Mexico City, Mexico; 3 Universidad Nacional Autónoma de México (UNAM), Mexico City, Mexico

## Abstract

**Background:**

Hepatocellular carcinoma is the most common cause of primary liver neoplasms and is one of the main causes of death in patients with liver cirrhosis. High Alpha fetoprotein serum levels have been found in 60–70% of patients with Hepatocellular carcinoma; nevertheless, there are other causes that increase this protein. Alpha fetoprotein levels ≥200 and 400 ng/mL in patients with an identifiable liver mass by imaging techniques are diagnostic of hepatocellular carcinoma with high specificity.

**Methods:**

We analysed the sensitivity and specificity of the progressive increase of the levels of alpha fetoprotein for the detection of hepatocellular carcinoma in patients with liver cirrhosis. Seventy-four patients with cirrhosis without hepatocellular carcinoma and 193 with hepatic lesions diagnosed by biopsy and shown by image scans were included. Sensitivity and specificity of transversal determination of alpha fetoprotein ≥ 200 and 400 ng/mL and monthly progressive elevation of alpha fetoprotein were analysed. Areas under the ROC curves were compared. Positive and negative predictive values adjusted to a 5 and 10% prevalence were calculated.

**Results:**

For an elevation of alpha fetoprotein ≥ 200 and 400 ng/mL the specificity is of 100% in both cases, with a sensitivity of 36.3 and 20.2%, respectively. For an alpha fetoprotein elevation rate ≥7 ng/mL/month, sensitivity was of 71.4% and specificity of 100%. The area under the ROC curve of the progressive elevation was significantly greater than that of the transversal determination of alpha fetoprotein. The positive and negative predictive values modified to a 10% prevalence are of: 98.8% and 96.92%, respectively; while for a prevalence of 5% they were of 97.4% and 98.52%, respectively.

**Conclusion:**

The progressive elevation of alpha fetoprotein ≥7 ng/mL/month in patients with liver cirrhosis is useful for the diagnosis of hepatocellular carcinoma in patients that do not reach αFP levels ≥200 ng/mL. Prospective studies are required to confirm this observation.

## Background

Hepatocellular carcinoma (HCC) is the most common cause of primary liver neoplasms and the fourth most frequent type of cancer worldwide with an increasing incidence, causing one million deaths *per *year[1]. Nowadays, therapeutic options are still not efficient, reaching a global survival rate of 3–10% at 5 years after diagnosis. The major risk factors associated to the development of HCC are infection with hepatitis B virus (HBV), hepatitis C virus (HCV) and cirrhosis of any cause[2]. HCC is one of the main causes of death in patients with Liver Cirrhosis (LC)[3]. The annual risk to develop HCC in patients with LC is 5% (1–7%), with a published prevalence between 7.4 and 23% found in necropsies of this group of patients. Cirrhosis is present in 80–90% of patients with this type of cancer [3].

Alpha-fetoprotein (αFP) is a protein of fetal component produced during the embryonic period by the visceral endoderm of the gestational sac and, later on, by the liver. Its re-expression in patients with HCC has been described for over 40 years. Some studies have demonstrated that the presence of elevated levels of αFP in patients with LC is a risk factor for the development of HCC [4-6], thus suggesting that increased αFP-production in patients with LC might reflect, largely and abnormal or altered liver cell regeneration. High αFP serum levels have been found in 60–70% of patients with HCC; nevertheless, there are other causes of increased levels, such as cirrhosis, lung cancer, biliary cancer, gastric cancer, pancreatic cancer, teratocarcinoma of the testis, spherocytosis and tyrosinemia[7]. Alpha fetoprotein levels <20 ng/mL are considered normal; with this value as the upper level, this diagnostic test has a sensitivity of 41–65%[4,8,9] to diagnose HCC, but a low specificity. The αFP levels diagnostic for HCC are above 400 ng/mL[10] in patients with LC and a solid liver mass >2 cm with typical features in one imaging study. The Italian and the American Association for the Study of Liver Diseases guidelines consider a level ≥200 ng/mL as the cut-off point for diagnosis [8,9,11-13]. Higher levels of αFP are associated with poor prognostic and survival rates in untreated patients with HCC or those treated with liver transplantation or locoregional therapies[4,8,14]. Some experts use αFP determinations and liver ultrasound every 3 to 6 months to detect HCC in patients with liver disease[15-17].

There is no concluding evidence available of the changes of αFP levels throughout the disease yet[18]. The aim of our study was to evaluate the progressive elevation of αFP as a diagnostic test in patients with LC with an hepatic lesion suspicious of HCC, and compare it to the transversal determination of αFP ≥200 and 400 ng/mL.

## Methods

This study was carried out in a single reference center (Instituto Nacional de Ciencias Médicas y Nutrición "Salvador Zubirán", National Institute of Medical Sciences and Nutrition) the patients were studied in the time period comprehended between January 1992 and December 2002, in the out-patient clinics of the Internal Medicine and Gastroenterology services. Patients with the diagnosis of LC and serial monthly determinations of αFP levels, were included; the presence of cirrhosis was established by biopsy or clinical data of chronic liver disease, complications such as portal hypertension, esophageal varices with or without a previous episode of bleeding, splenomegaly, ascites with a previous episode of spontaneous bacterial peritonitis in the absence of other non hepatic causes, hepatic encephalopathy in the absence of other metabolic causes, hypoalbuminemia or hyperbilirubinemia in the absence of a known cause of the obstruction of the bile duct of at least one year of appearance with progressive liver failure. All patients in the control group had a minimal follow-up of one year with monthly determination of serum αFP levels and imaging studies every 3 to 6 months in order to assess they did not have HCC. Ultrasound, Computed Tomography and Magnetic Resonance Imaging were the diagnostic studies employed in this work, the use of one or another depended on the treating physicians' personal preferences.

Patients who were diagnosed with HCC by biopsy and had monthly determinations of αFP levels were included as cases. Patients in which the diagnosis of HCC was performed by other means (e.g. alternative methods of imaging diagnosis) were excluded. Files from the clinical archive were reviewed. The exclusion criteria were: patients with incomplete files, patients who did not meet diagnosis criteria for LC or HCC, patients who were misdiagnosed, or who did not have at least three serial monthly determinations of the levels of αFP reported. A database was done registering clinical and pathological information. All patients were classified based on the Child-Pugh-Turcotte criteria (based on serum bilirubin and albumin, ascites, neurological disorder and nutrition), which are established prognostic factors in patients with liver cirrhosis undergoing surgery [19,20].

The comparison of the clinical and pathological characteristics between patients with and without an elevation of αFP ≥ 200 and 400 ng/mL, as well as between those with and without progressive elevation was done with student's T test and Mann-Whitney's U for continuous variables with and without normal distribution, respectively. For comparisons between more than three variables, ANOVA and Tukey correction tests were used. Qualitative variables were compared with Chi squared and exact Fisher's test. A p value <0.05 was considered as significant.

Sensitivity (i.e. True Positive Rate) and specificity (i.e. True Negative Rate) were obtained by determining the levels of αFP and its progressive elevation (the average monthly increase of at least three measurements was required), using SPSS statistical software version 10.0.

The Receiving Operating Characteristic (ROC) Analysis curves and the corresponding area under the curve were calculated to provide the accuracy of serum αFP in differentiating HCC and LC patients. Non-parametric estimates of the area under the ROC curve and the respective standard error were applied. Positive (PPV) and Negative (NPV) predictive values were evaluated in our study considering a prevalence of HCC of 5 and 10%. This was done with EPIDAT software version 3.

The experimental research reported in this manuscript has been performed with the approval of the Institutional Review Board and Ethics Committee of the National Institute of Medical Sciences and Nutrition and is in compliance with the Helsinki Declaration.

## Results

We obtained 212 files of patients with the diagnosis of HCC and 202 of patients with LC; from which 193 and 74 patients were included, respectively. The main causes of exclusion were: incomplete files, lack of αFP determinations, and an ambiguous diagnosis. No differences were found among the patients considered as controls and those excluded with LC regarding female gender (60 vs 63%, p = 0.9), age (51. 7 ± 1.7 vs 50.7 ± 2 years; p = 0.8), Child (A/B/C 40/42/18 vs 55/34/11%, p = 0.4) and etiology (alcoholic 11.2 vs 14, HCV 45 vs 50, cryptogenic 32 vs 15.9 p = 0.5), serum αFP levels (8.2 ± 2.2 vs 13 ± 4.4 ng/mL, p = 0.3). This analysis included only 85 of the excluded patients due to an insufficient accuracy of the medical files.

The minimal follow-up time of the patients with LC without HCC (control cases) was of one year. The median follow-up time of the patients with HCC was of 4.5 ± 1.2 months. Seventy-five percent of the patients with HCC had a tumor size >5 cm. Tumors were multicentric in 22% and poorly differentiated in 35%. General characteristics of patients with HCC and LC are described in Table 1. We found a greater proportion of men and a greater mean age for patients with HCC. No differences regarding the Child-Pugh score were found between groups. The αFP level was significantly higher for patients with HCC compared to patients with LC (8.2 ± 2.2 vs 271 ± 46 ng/mL, respectively, *p *< 0.001). The progressive elevation of αFP was greater in patients with HCC when compared to patients with LC (0.34 ± 0.2 vs 62.3 ± 13.5 ng/mL/month, respectively, *p *< 0.001) (Table 1). No differences were found between the αFP elevation and the Child-Pugh score, age, gender, etiology, differentiation grade and tumour size amongst patients with HCC (Table 2). All patients of both groups have at least 3 determinations of αFP. The median number of αFP determinations was of 3, range from 3 to 5 in both groups. The average increase of the levels of αFP in patients with αFP basal levels <20 ng/mL was of 15.8 ng/mL ± STD error 10.7, 67.9 ng/mL ± 38 in the patients with a basal αFP level between 20 and 100 ng/mL, and 76.3 ng/mL ± 18 for the patients with αFP levels >200 ng/mL (p = 0.211). An elevation of αFP ≥7 ng/mL/month in one determination was observed in 16.4% and 70.8% of patients with LC and HCC, respectively; while 1.8% and 43.2% of patients with LC and HCC showed an elevation ≥7 ng/mL/month in two determinations.

**Figure 1:**
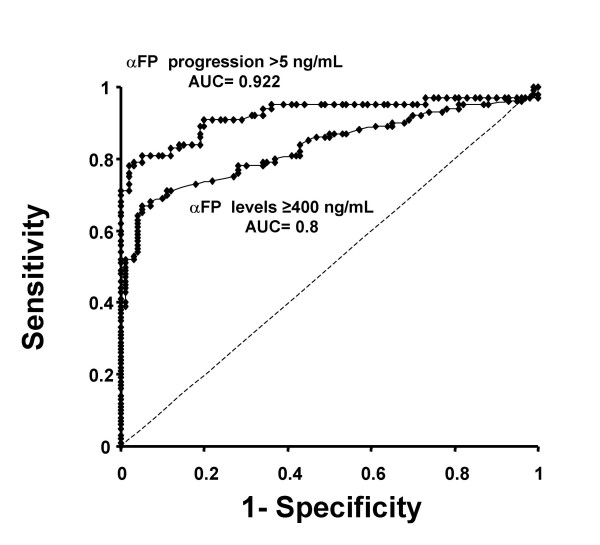
ROC curve for the progressive elevation of αFP and αFP levels. The area under the curve for the progressive elevation of αFP is significantly greater than the area under the curve for αFP levels (p < 0.05). (AUC = Area Under the Curve).

The sensitivity and specificity for the transversal determination of αFP varied according to its level as shown in Table 3. For an αFP elevation ≥ 200 and 400 the specificity is of 100% in both cases, with a sensitivity of 36.3 and 20.2%, respectively. The sensitivity and specificity for the progressive determination of αFP are shown in Table 4. In Figure 1, the areas under the ROC curves for the αFP levels and the progression of αFP are shown. The area is significantly greater for the αFP progressive elevation group (p < 0.05). In patients with an αFP progression rate ≥7 ng/mL/month a sensitivity of 71.4% and a specificity of 100% were found. We obtained an area under the curve of 0.587 with αFP levels ≥200 ng/ml, and 0.82 with the progressive elevation of αFP ≥ 7 ng/mL/month, *p *< 0.001.

For patients with baseline αFP levels >7–20, sensitivity, specificity, positive predictive value (PPV) and negative predictive values (NPV) were: 50, 100, 100 and 78.7%, respectively. For those with αFP baseline levels from 21 to 100, sensitivity, specificity, PPV and NPV were of 85, 100, 100 y 87.5%, respectively; between 101 and 200, these values were of 50, 100, 100 y 25%, respectively.

Table 5 reports a PPV and NPV of ≥200 and 400 ng/mL, as well as for the progressive elevation of αFP ≥7 ng/mL/month taking into account a prevalence of 5 and 10%. The positive and negative predictive values for the progressive elevation of αFP ≥7 ng/mL/month modified to a 10% prevalence are of: 98.8% and 96.92%, respectively; while for a prevalence of 5% they were of 97.4% and 98.52%, respectively. This PPV could be used to reveal the neoplastic nature of a liver mass occurring during surveillance in patients without a transversal elevation of αFP.

## Discussion

Many studies have evaluated the αFP serum levels as a diagnostic test for HCC in patients with LC[9]. Due to the poor prognosis of patients with HCC, in order to improve survival, the therapeutic approach mainly depends on an early diagnosis. Besides being a diagnostic tool, determination of αFP has also been used to evaluate the response to treatment and, moreover, to detect recurrences[10,21]; other studies have demonstrated that elevated levels of αFP are an independent risk factor for the development of HCC in patients with LC[5,6].

The causes of liver disease in our study were: alcoholic liver disease, infection with either hepatitis B or hepatitis C viruses, and cryptogenic (Table 1), as previously reported; but with a different frequency[22,23]. Studies have found differences in the αFP levels related to: the tumoral size and tumor doubling time the Child-Pugh score and the age of patients with HCC[21,24,25]; however this was not corroborated either in our study, or by other authors[26-28]. Even though the patients in our study had a close follow-up, 75% were found to have large tumors (>5 cm). Although there are studies suggesting that an intensive surveillance program may identify smaller tumors, this is still controversial [29,30]. Furthermore, other studies have not found a statistically significant relationship between the positivity of αFP and tumoral size, degree of vascular invasion and the histological differentiation grade of the tumor. Nevertheless, patients with positive staining for αFP had a worse prognosis than those with negative staining [31].

The sensitivity and specificity of αFP for the detection of HCC has been reported from 17 to 76% and 80 to 100%, respectively (Table 6)[8,18,32-36]. We found that an αFP value ≥200 and 400 ng/mL has a sensitivity of 36.3% and 20.2%, respectively, and specificity of 100% in both groups; a study with 340 patients found similar results[18]. There can be a transitory elevation of αFP in patients with cirrhosis and exacerbations of infectious hepatitis[37]. Alpha-fetoprotein levels are not elevated in many patients with HCC, which makes the transversal measuring of the αFP elevation not an optimal diagnostic test. Moreover the clinical usefulness of alpha-fetoprotein in hepatocellular carcinoma management is still being debated [38].

We observed that an αFP monthly average progression rate ≥7 ng/mL/month of at least 3 determinations, has a sensitivity and specificity of 71.4 and 100%, respectively. The elevation of αFP after one month of follow-up sometimes did not show any changes; we realized that 3 determinations were required in order to have a more accurate test.

The αFP progression rate and a tumor-suggestive image – together – could be employed to diagnose HCC. This approach could be of special interest in patients with LC and clinical suspicious of HCC, who are not candidates neither for biopsy nor for surgical treatment and who do not reach an αFP level ≥200 ng/mL; they could benefit of being diagnosed with HCC and initiate treatment without waiting for αFP levels to reach a value ≥ 200 or ≥400 ng/mL. Many patients with a suggestive image of HCC can not be biopsied due either to coagulation abnormalities or location of the tumor or due to the presence of massive ascites among other causes; also, many patients do not reach the value of 200 or 400 ng/mL (in our study 63.7% and 79.8%, respectively), reason why the progressive elevation of αFP could help to establish the diagnosis without the need of biopsy and could allow the inclusion of these patients to treatment study protocols. On the other hand, the continuous increase of aFP should induce the start of research protocols for HCC and eventually enrollment of these patients in a transplantation program even before the tumor becomes detectable.

There are few existing studies that evaluate the utilization of serial determinations of αFP for the diagnosis of HCC and none compares it to the transversal determination of αFP at its best specificity level (≥ 200 ng/mL)[18,39,40]. A study demonstrated that a fast and sudden elevation of αFP in patients with liver lesions detected more HCC when compared to other elevation patterns in patients with cirrhosis and hepatic lesions[41]. Many studies have been done to increase the specificity of the measurement of αFP for the diagnosis of HCC depending on its binding capacity to different molecules; however its use is expensive and it is not widely available[2,42].

Even though high αFP levels may be more indicative of HCC in patients with LC without viral infection [9], the type of viral infection (HBV or HCV) does not seem to have a direct influence on the serum αFP levels in patients with HCC [21,43]. A limitation of this study are the differences in etiology between both study groups, mainly regarding HBV patients, which could cause the differences in the αFP levels. Also, since it is a retrospective study, it was not possible to match both groups from the beginning. Prospective studies need to be done in order to corroborate our findings and to perform a cost-benefit analysis of this test. Nevertheless, these studies might be difficult to perform; as a matter of fact, if we consider that approximately 5% of the patients with viral hepatitis and LC develop HCC within a year, the number of patients with LC needed to carry out such studies is rather large.

## Conclusion

In conclusion, the average progressive elevation of αFP ≥7 ng/mL/month and a tumor-suggestive image – together – in patients with LC could be a very useful tool for the detection of HCC, especially in those patients without a transversal αFP elevation ≥200 ng/mL.

## Competing interests

The author(s) declare that they have no competing interests.

## Authors' contributions

BC, AVR, NHP and DME reviewed medical records and participated in the design. DME helped to draft the manuscript and contributed to the statistical analysis. OA conceived of the study, and participated in its design and coordination, performed the statistical analysis and helped to draft the manuscript. All authors read and approved the final manuscript.

## Pre-publication history

The pre-publication history for this paper can be accessed here:



## Figures and Tables

**Table 1:** General characteristics of the patients with liver cirrhosis (LC) and hepatocellular carcinoma (HCC)

Characteristics	LC	HCC	*P*
Female	60%	46%	0.049
Male	40%	64%	
			
Age	51.7 ± 1.7	59.9 ± 0.96	0.001
			
Child-Pugh Score			
A	40%	47%	
B	42%	37.3%	0.48
C	18%	15%	
			
Etiology			
Alcoholic	11.2%	26%	
HBV	0%	7.2%	
HCV	45%	30%	0.001
Cryptogenic/autoimmune	32%	5.7%	
Undetermined or absent	11%	30.4%	
			
αFP	8.2 ± 2.2	271 ± 46	0.001
			
αFP > 400	0%	19.6%	0.001
			
Progressive monthly elevation	0.34 ± .161	62.3 ± 13.5	0.001
			
Increase of 5 ng/mL/month or greater	1.7%	76.2%	0.001

**Table 2:** Clinical and pathological factors associated with the levels of αFP in patients with hepatocellular carcinoma (HCC).

Associated Factors	Levels of FP α(ng/mL)	*P*
Child-Pugh Score		
A	171 ± 34	0.74
B	229 ± 73	
C	211 ± 80	
Mean age		
<60 years	267 ± 64	0.78
>60 years	132 ± 19	
Gender		
Female	164 ± 43	0.49
Male	238 ± 53	
Etiology		
HBV	368 ± 36	0.16
HCV	137 ± 23	
Cryptogenic/autoimmune	52 ± 23	
Alcoholic	275 ± 99	
Undetermined or absent	258 ± 90	
Degree of differentiation		
Well	167 ± 29	0.16
Moderate	271 ± 87	
Poor	140 ± 10	
Tumor Size		
< 5 cm	267 ± 64	0.66
> 5 cm	132 ± 19	

**Table 3:** Sensitivity and Specificity for the transversal determinations of αFP for the diagnosis of hepatocellular carcinoma (HCC)

αFP (ng/mL)	Sensitivity (%)	Specificity (%)
0.3	99.5	0
2.5	92.2	29.7
5.1	83.9	57
7.5	77.7	72
10.2	71	88
15.5	64.2	96
20.3	60.6	96
25.6	58.5	96
51.35	51.8	99
72.5	48.7	99
98.65	47.2	99
111	45.6	99
130	43	99
160	38.9	100
190	36.3	100
215	34.7	100
280	24.4	100
330	21.8	100
395	20.2	100
415	18.7	100

**Table 4:** Sensitivity and Specificity for the progression rate of αFP

Progression rate (ng/mL/month)	Sensitivity (%)	Specificity (%)
-3.43	100	0
0.1065	95.2	56
0.2335	92.1	70
0.66	87.3	82
0.94	84.1	85
1.6	81	89
2.15	81	94
2.64	81	95
3.01	79.4	95
3.29	79.4	97
4.04	77.8	97
4.75	77.8	99
5.07	76.2	99
5.47	74.6	99
5.84	71.4	100
8.87	66.7	100
10.8	65.1	100
16.83	52.4	100
20.4	46	100
28.94	38.1	100

**Table 5:** Positive Predictive Value (PPV) and Negative Predictive Value (NPV) for the diagnosis of HCC with αFP values ≥200, αFP values ≥ 400 ng/mL and with the progressive elevation of αFP of 5 ng/mL with a prevalence of 5 and 10%.

**αFP**	**HCC Prevalence (%)**	**PPV (%)**	**NPV (%)**
≥200 ng/ml	10	97.58	93.4
	5	95.03	96.7
≥400 ng/ml	10	95.7	91.86
	5	91.4	95.97
Elevation ≥7 ng/ml/month	10	98.7	96.92
	5	97.4	98.52

**Table 6:** Comparison of sensitivity and specificity of the αFP levels for the diagnosis of hepatocellular carcinoma (HCC) of several studies

Authors	αFP 10–16 (ng/mL)		αFP 20 (ng/mL)		αFP 100 (ng/mL)		αFP 200 (ng/mL)		αFP 300 (ng/mL)		αFP 400 (ng/mL)	
	S (%)	E (%)	S (%)	E (%)	S (%)	E (%)	S (%)	E (%)	S (%)	E (%)	S (%)	E (%)

Oka *et al *1994			39	76	13	97						
Pateron *et al *1994	50	86			21	93						
Peng *et al *1999			65	87			45	100				
Tong *et al *2001			41	94								
Trevisani *et al *2001	62.4	89.4	60	90.6	31.2	98.8	22	99	22.4	99.4	17.1	99.4
Gebo *et al *2002*			60								0–64	100
Nguyen *et al *2002	78.4	61.1	63	80	41.4	97.3	32	100				
Gupta *et al *2003*			41–65	80–94								
Farinati *et al *2006			54								18	
Arrieta 2007^+^	63.2	95.9	60.6	95.9	47.2	99	36.3	100	22.8	100	20.2	100

S = sensitivity, E = specificity.

* Literature review

^+ ^Present study
